# An unexpected pattern of tracer uptake on pulmonary ventilation/perfusion SPECT imaging: a case report

**DOI:** 10.1093/ehjcr/ytag361

**Published:** 2026-05-13

**Authors:** Teemu Maaniitty, Tommi Noponen, Antti Saraste, Marko Seppänen

**Affiliations:** Turku PET Centre, Turku University Hospital and University of Turku, P. O. Box 52, Turku 20521, Finland; Department of Clinical Physiology, Nuclear Medicine and PET, Turku University Hospital, P. O. Box 52, Turku 20521, Finland; Department of Clinical Physiology, Nuclear Medicine and PET, Turku University Hospital, P. O. Box 52, Turku 20521, Finland; Department of Medical Physicists, Turku University Hospital, P. O. Box 52, Turku 20521, Finland; Turku PET Centre, Turku University Hospital and University of Turku, P. O. Box 52, Turku 20521, Finland; Heart Center, Turku University Hospital, P. O. Box 52, Turku 20521, Finland; Department of Clinical Physiology, Nuclear Medicine and PET, Turku University Hospital, P. O. Box 52, Turku 20521, Finland

## Case description

A 66-year-old woman was referred to pulmonary ventilation/perfusion single photon emission computed tomography (SPECT) due to persistent exercise intolerance and hypoxaemia. Ventilation SPECT images demonstrated homogeneous pulmonary tracer uptake (*Figure 1A*). Subsequent SPECT images obtained after the administration of a perfusion tracer (technetium-99m macroaggregated albumin, A = 150 MBq) via a venous cannula placed on the patient’s left wrist showed an unexpected uptake of the tracer in the kidneys and spleen (*Figure 1B*). Moreover, pulmonary counts on the perfusion scan were slightly lower than the counts on the ventilation scan, indicating the absence of perfusion tracer uptake in the lungs. This extrapulmonary tracer distribution reflects right-to-left shunting and systemic embolization of the perfusion tracer. Pulmonary perfusion SPECT was repeated on a separate day, this time with tracer injected via a venous cannula placed on the patient’s right arm to prevent shunting, demonstrating a normal pattern of pulmonary perfusion tracer uptake (*Figure 1C*).

**Figure 1:**
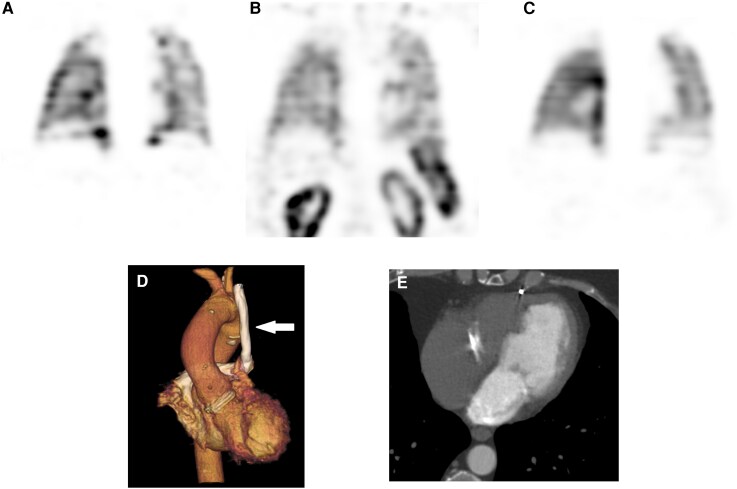
Ventilation single photon emission computed tomography (SPECT) images demonstrated homogeneous pulmonary tracer uptake (*A*). Subsequent perfusion SPECT images showed unexpected tracer uptake in the kidneys and spleen (*B*). This was caused by a persistent left superior vena cava that anomalously drained into the heart’s left atrium via the left upper pulmonary vein, resulting in right-to-left shunting and systemic embolization of the perfusion tracer, which had been administered via a venous cannula placed on the patient’s left wrist. The persistent left superior vena cava with anomalous drainage (arrow in *D*) was confirmed by computed tomography scan with iodine contrast agent administered into a vein of the left arm, showing the absence of contrast agent on the right-sided cardiac chambers (*E*). Perfusion SPECT imaging was repeated on a separate day, this time with tracer injected via a venous cannula placed on patient’s right arm to prevent shunting, demonstrating normal pattern of pulmonary perfusion tracer uptake (*C*).

Due to the suspicion of shunting, the patient subsequently underwent computed tomography with contrast agent administered into a vein of the left arm, demonstrating a persistent left superior vena cava (arrow in *Figure 1D*) that anomalously drained into the heart’s left atrium via the left upper pulmonary vein, and hence, resulted in right-to-left shunting. Contrast agent was absent on the right-sided cardiac chambers (*Figure 1E*). Cardiac magnetic resonance imaging demonstrated pulmonary-systemic flow ratio of 0.4, indicating a haemodynamically significant right-to-left shunt. The patient was treated with a successful percutaneous endovascular closure of the anomalous connection.

The estimated prevalence of persistent left superior vena cava is 0.2%–3% in the general population, with only 10%–20% of these cases showing drainage into the left atrium.^1^ The unexpected pattern of tracer uptake on pulmonary ventilation/perfusion SPECT raised the suspicion of shunting in this patient, whereas additional imaging modalities were needed to confirm and further characterize this. On SPECT imaging, ventilation and perfusion counts should always be compared to ensure adequate perfusion tracer uptake in the lungs. Furthermore, injection site selection has relevance in studying the aetiology and physiology of suspected shunting and requires special attention in patients with a known shunt.

## Data Availability

The data underlying this article are available in the article.

## Notes

*Consent.* The authors confirm that written consent for submission and publication of this case report including images has been obtained from the patient in line with the COPE guidance.
